# Recent efforts of vapour-phase strategies for EUV resist toward high- and hyper-NA extreme ultraviolet lithography

**DOI:** 10.1039/d6sc02112c

**Published:** 2026-06-10

**Authors:** Thi Thu Huong Chu, Dan N. Le, Minjong Lee, Dushyant M. Narayan, Doo San Kim, Soham Shirodkar, Minki Choe, Suyoung Yoo, Nikhil Tiwale, Rino Choi, Jinho Ahn, Myung Mo Sung, Chang-Yong Nam, Jiyoung Kim

**Affiliations:** a Department of Materials Science and Engineering, The University of Texas at Dallas Richardson Texas 75080 USA Jiyoung.Kim@utdallas.edu; b Department of Electrical and Computer Engineering, The University of Texas at Dallas Richardson Texas 75080 USA; c Department of Materials Science and Engineering, Inha University Incheon 22212 Republic of Korea; d Program in Environmental and Polymer Engineering, Inha University Incheon 22212 Republic of Korea; e Center for Functional Nanomaterials, Brookhaven National Laboratory Upton New York 11973 USA; f Department of Materials Science and Engineering, Hanyang University Seoul 04763 Republic of Korea; g Department of Chemistry, Hanyang University Seoul 04763 Republic of Korea; h Department of Materials Science and Chemical Engineering, Stony Brook University Stony Brook New York 11794 USA

## Abstract

Extreme ultraviolet lithography (EUVL, *λ* = 13.5 nm) is critical for sub-1 nm technology nodes but remains constrained by inherent trade-offs among resolution, line-edge roughness (LER), and sensitivity. Stochastic effects originating from photon shot noise, low-energy secondary electron blur, and the random distribution of resist components further limit its advancement toward high numerical-aperture (NA, NA = 0.55) and hyper-NA (NA ≥0.75) EUVL. While the optimization of spin-on chemically amplified resists (CARs) continues, metal-oxide resists (MORs) have emerged as strong candidates for next-generation EUVL by incorporating metals with high EUV absorption coefficients, which enhances both resist sensitivity and etch resistance during pattern transfer. Besides spin-coating, recent advances in vapour-phase techniques, such as vapour-phase infiltration (VPI), chemical vapour deposition (CVD), and molecular atomic layer deposition (MALD), offer promising pathways to achieve new resist platforms, such as dry resists, that satisfy the stringent thickness and uniformity requirements of next-generation EUVL. These methods enable the direct incorporation of metal species into existing resist matrices or the formation of hybrid inorganic–organic resist platforms, thereby improving film uniformity, etch durability, and pattern fidelity while mitigating stochastic defects. This review highlights the latest advancements in vapour-phase-synthesized EUV resists, emphasizing material design, lithographic performance, and the underlying exposure mechanisms. Although still emerging, vapour-phase strategies are paving the way for an all-dry integration framework that could improve EUV patterning workflows and meet the demands of future technology nodes.

## Abstract 2

This review highlights recent progress in vapour-phase strategies for EUV resists, emphasizing dry resist design and solvent-free development toward high-fidelity patterning for high- and hyper-NA lithography.

## Graphical abstract

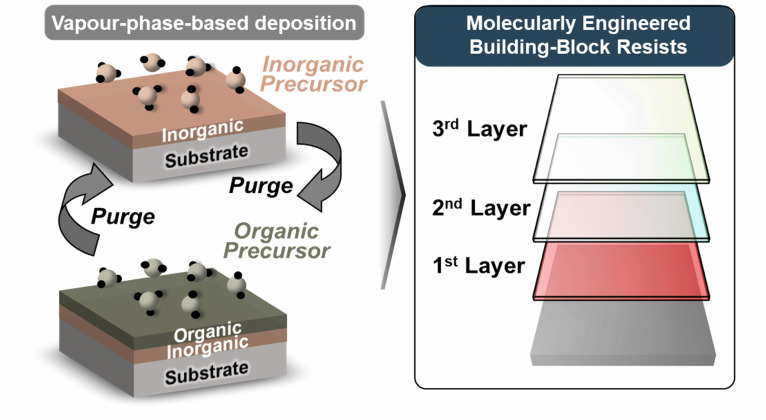

## Introduction

**Fig. 1:**
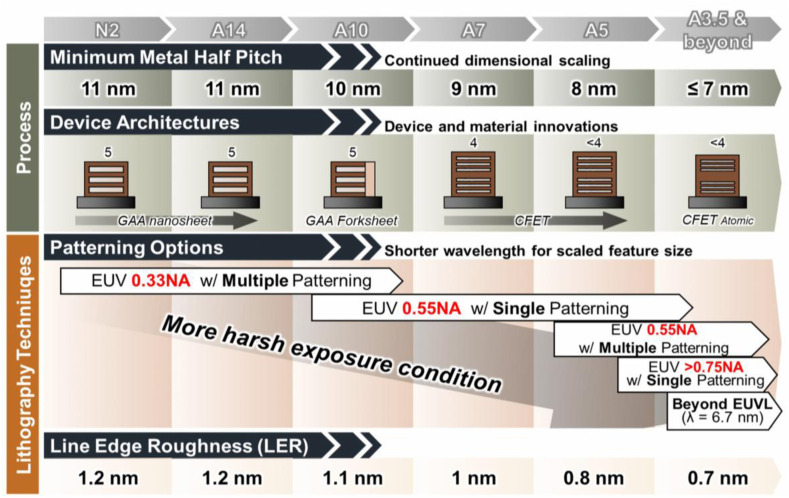
Semiconductor logic node scaling roadmap highlighting the evolution of lithography trends. Adapted from ref. 1 and 2.

**Fig. 2:**
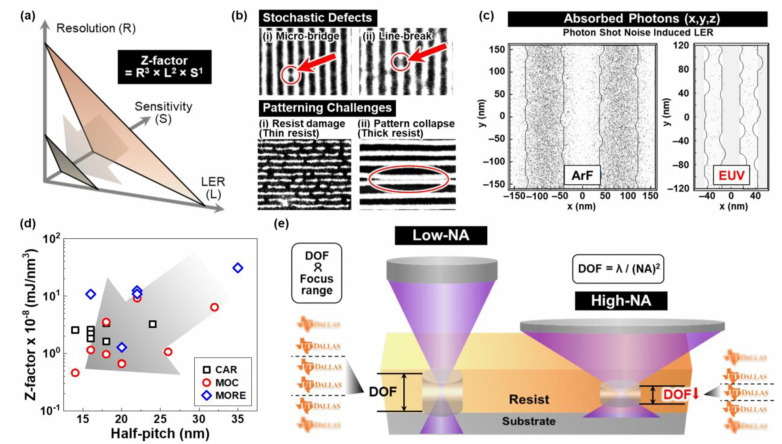
Key challenges in advanced EUV lithography. (a) A schematic illustration of resolution (R), line-edge roughness (LER), and sensitivity (S) trade-off. The overall performance of a photoresist is frequently benchmarked using the *Z*-factor. (b) Stochastic defects (*e.g.*, micro-bridges and line breaks) and patterning issues in thin and thick resists lead to challenges such as poor pattern transfer and pattern collapse, respectively. (c) Photon shot noise-induced LER in ArF (193 nm) *versus* EUV (13.5 nm) lithography, highlighting the more pronounced stochastic effects in EUV arising from its inherently lower photon absorption density relative to ArF lithography.^17^ Reproduced with permission from SPIE. (d) Reported *Z*-factor values for various EUV photoresists as a function of half-pitch (HP). (CAR: Chemically Amplified Resists; MOC: Metal-Oxo Clusters; MORE: Molecular Organometallic Resists). Data adapted from ref. 32. (e) Depth-of-focus reduction with increasing numerical aperture (NA).

Extreme ultraviolet (EUV, *λ* = 13.5 nm) lithography has emerged as a transformative technology for advanced semiconductor manufacturing, enabling patterning of features below 10 nm and extending the scaling roadmap toward sub-nm nodes, as shown in Fig. 1.^1,2^ The push toward smaller critical dimensions (CDs) is driven by the ever-increasing demand for higher transistor density, improved performance, and reduced power consumption in modern integrated circuits (ICs).^3,4^ As a result, increasingly complex device architectures with smaller feature sizes have been adopted, necessitating more complex lithographic patterning procedures to achieve the required dimensions and patterning fidelity.^5,6^ However, in addition to the ongoing advancement of light sources and associated optics, the miniaturization of devices has placed unprecedented demands on photoresist materials that must simultaneously meet several competing requirements.^7^ In particular, achieving high resolution, low line-edge roughness (LER), and sufficient sensitivity remains a persistent challenge, known as the resolution-LER-sensitivity (RLS) trade-off (Fig. 2a).^8^

Since the development of deep ultraviolet (DUV, *λ* = 193 nm) lithography, chemical-amplified resists (CARs), consisting of polymer backbones, photoacid generators (PAGs), quenchers, solvents, and other components, have been extensively studied and remain the cornerstone of EUV lithography.^9^ Over the years, significant efforts have been devoted to improving the performance of CARs.^10–15^ For example, to enhance sensitivity, highly reactive components have been introduced to accelerate the reaction kinetics; however, this improvement comes with a trade-off, as increased stochastic effects lead to higher LER and the introduction of defects.^13^ These stochastic defects, including micro-bridging and line-breaks, ultimately reduce the final device yield at the smallest CDs, while inadequate pattern transfer in thin resists and pattern collapse in thick resists further complicate scaling (Fig. 2b).^16^

Notably, the stochastic effect induced by photon shot noise is more pronounced in EUV lithography due to the inherently lower photon absorption density compared with ArF DUV lithography, as demonstrated in Fig. 2c.^17^ The number of EUV photons absorbed at a given dose is approximately 14 times lower than that of 193 nm photons under equivalent NA conditions, owing to the higher photon energy and lower photon flux of EUV radiation.^18^ The limited absorption of photons amplifies stochastic variations, leading to higher LER that causes variations in circuit element dimensions and overlay errors, resulting in a degraded performance of ICs.^19,20^ According to the technology roadmap, the target LER must be reduced to 0.7 nm by 2035, corresponding to the 3.5 Å node.^1^ However, as initial EUV resists, CARs exhibit significant LER of approximately 2.5 nm, mainly due to acid-diffusion-induced blurring.^21–24^ Despite extensive efforts, including the introduction of quenchers and multi-trigger systems requiring multiple activation events for chemical transformation, the LER remains relatively high (>1.7 nm).^25,26^

Recognizing the inherent limitation of traditional resist materials, alternative metal-oxide resists (MORs) have been explored, including metal-oxo clusters (MOCs) and molecular organometallic resists (MOREs).^27–30^ In these systems, highly EUV-absorbing metal elements are incorporated to improve both sensitivity and etch resistance.^30^ Although these approaches have demonstrated improved lithographic performance, post-development residues remain a major challenge for such materials. The *Z*-factor, defined by the equation in the inset of Fig. 2a, is widely used as a key figure of merit for assessing photoresist performance.^31^ A lower *Z*-factor indicates better patterning performance, characterized by higher resolution, reduced LER, and improved sensitivity. Fig. 2d summarizes the reported *Z*-factor for three EUV resist platforms, including CARs, MOCs, and MOREs, plotted as a function of the minimum well-resolved half-pitch (HP).^32^ The trend clearly reveals that incorporating metal elements enhances EUV photon absorption and overall resist efficiency, thereby lowering the *Z*-factor. These results highlight the crucial role of metal-containing components in attaining the sensitivity, etch resistance, and pattern fidelity necessary for next-generation EUV lithography.

Furthermore, the shift from low-NA (NA = 0.33) to high-NA (NA = 0.55) EUVL is driven by the demand for continued CD scaling, where CD = *k*_1_(*λ*/NA), with *k*_1_ being a dimensionless process factor (typically <0.5) that is affected by optical components, exposure conditions, photoresist, and so on.^31,33,34^ This advancement is accompanied by a significant reduction in the depth of focus (DOF) inherent to EUV optical projection systems, as described by the following equation DOF = *k*_2_(*λ*/NA^2^) with *k*_2_ being another process factor specific to DOF.^31^ This means that when transitioning from 0.33 NA to 0.55 NA, the DOF reduces by approximately 2.8×, while the resolution improves by about 0.6×. A further increase to 0.75 NA (hyper-NA) would result in an estimated 5.2× reduction in DOF, with a corresponding 0.44× improvement in resolution. These values are estimated assuming equivalent *k*_1_ and *k*_2_ factors, thereby isolating the impact of NA scaling alone. However, the reduced DOF imposes stringent requirements on resist film thickness, necessitating sub-10 nm resists to maintain acceptable imaging performance, as thicker films exacerbate sensitivity to defocus, CD variation, and stochastic variability (Fig. 2e).^35,36^ Besides challenges in achieving uniform coating across a large substrate area, conventional spin-coated resists at such reduced thicknesses suffer from significant inhomogeneity, including variations in free volume, polymer entanglement, and residual solvent, which ultimately affects photon absorption and EUV-generated secondary electron blur, thereby compromising pattern fidelity and LER.^37,38^ These limitations underscore the need for innovative material design and processing strategies that can meet the stringent requirements of next-generation EUV lithography.

In response, vapour-phase strategies, including resist fabrication and development approaches, have recently emerged as promising pathways to overcome the limitations of conventional spin-coated resists. For resist fabrication, vapour-phase synthesis approaches such as chemical vapour deposition (CVD) and derivatives of atomic layer deposition (ALD), such as vapour-phase infiltration (VPI) and molecular atomic layer deposition (MALD), enable molecular-level control over film growth, composition, and bonding environments.^18,39^ Through careful tuning of precursor chemistry, pulse sequence, and process temperature, the metal-to-organic ratio and coordination structure within the film can be precisely engineered.^40–42^ This level of compositional control is crucial as it can directly influence the generation, transport, and dissipation of secondary electrons (SEs) following EUV photon absorption. It is well-known that upon EUV photon absorption, low-energy secondary electrons (SEs) are generated and subsequently interact with the resist film, driving the chemical transformations responsible for solubility changes.^43–47^ Building upon this fundamental understanding, electron beam lithography (EBL) is commonly employed to evaluate EUV resist materials. The use of EBL as a proxy for EUV exposure has proven effective, as it mimics aspects of secondary electron generation that represent the major interaction between EUV photons and photoresists.^48^ However, it should be noted that EUV photon absorption is governed by absorption cross-sections, whereas electron-induced interactions are determined by scattering cross-sections, leading to differences in initial ionization pathways. In addition, EUV photon absorption directly excites molecules into highly excited electronic states with distinct internal energy distributions, whereas electron impact ionization results in different excitation conditions. Despite these differences, EBL remains a valuable comparative tool for assessing resist response trends. Although some studies have employed EBL at energies far exceeding those of EUV photons, the secondary electrons generated during such high-energy exposures typically possess kinetic energies around 100 eV. Electrons within this low-energy regime exhibit higher interaction cross-sections with resist molecules, so most electron-induced chemical transformations are driven by these secondary electrons rather than the primary beam. While differences in the secondary-electron energy distributions between EUV and EBL exposures can lead to distinct reaction pathways, comparative studies of organic resists under both conditions have demonstrated a strong correlation in their sensitivities. This finding suggests that, for certain resist materials, EBL can reproduce the key chemical processes that occur during EUV exposure, making it a reliable method to rapidly evaluate EUV resists.

Beyond vapour-phase resist synthesis, dry development processes have also emerged as a prominent strategy for high-NA and hyper-NA EUV lithography.^49^ Unlike conventional wet development, which relies on solvents to dissolve exposed resist regions, dry development employs gaseous etchants or plasma to selectively remove material, enabling truly solvent-free patterning.^49,50^ This eliminates solvent-induced swelling, resists dissolution variability and pattern distortion, all factors that are major contributors to LER and line collapse in nanoscale features.^51,52^ When combined with vapour-phase-synthesized resists, dry development enables a fully solvent-free lithographic workflow that has attracted interest for future EUV lithography due to its potential to mitigate pattern collapse and improve pattern fidelity during high-resolution patterning.

In this review, we discuss recent advances in vapour-phase strategies for developing new photoresists for EUV lithography, with a focus on the design of dry resist materials and solvent-free development processes that enhance pattern fidelity. By consolidating progress in these approaches and their application to advanced resist platforms, this review aims to guide researchers in the rational design of fully dry EUV lithography materials. Integrating chemical control with process engineering is essential to overcome the inherent limitations of conventional resist systems and to enable continued scaling in semiconductor manufacturing.

## Current progress on vapour-phase synthesized resists for high-NA and hyper-NA EUVL

### Chemical vapour deposition (CVD)

CVD is a widely used thin-film deposition technique in the semiconductor industry, particularly for fabricating semiconducting, insulating, and metal films.^53^ In this process, volatile precursor gases are introduced into a reaction chamber, where they react or thermally decompose upon contact with a heated substrate surface.^54^ This reaction results in the formation of a uniform, solid thin film, with growth primarily governed by mass transport and surface reaction kinetics.

**Fig. 3:**
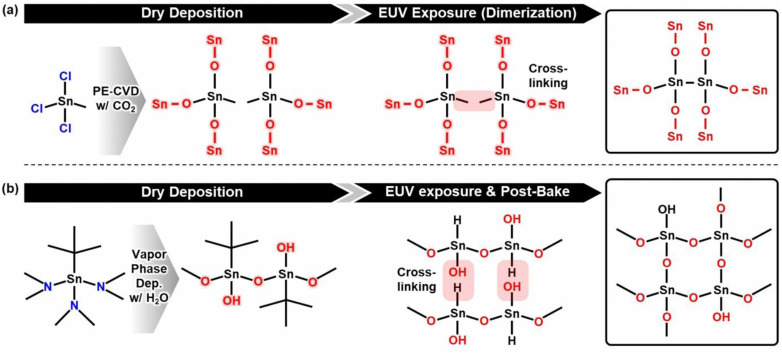
Schematic illustration of the dry deposition and EUV exposure processes for Lam Research's Sn-based resists: (a) PE-ALD-based Sn resist and (b) vapour-phase-deposited Sn resist.

The first several dry-deposited tin (Sn)-based resist systems were developed by Lam Research using thermal and plasma-enhanced CVD (PE-CVD) techniques. In 2017, Lam Research reported a PE-CVD process for Sn-based negative-tone resist thin films that employed tin precursors such as trimethyltin chloride [(CH_3_)_3_SnCl], reacted with plasma-activated oxidants, such as carbon dioxide (CO_2_).^55^ The plasma provides energetic radicals that promote low-temperature film growth and enable the formation of CH_3_Sn(SnO)_3_ networks. Upon EUV irradiation, the deposited organotin oxide thin film undergoes dimerization or condensation reactions, where cleavage of Sn–CH_3_ bonds leads to the formation of a cross-linked Sn_2_(SnO)_3_)_2_ network in the exposed regions (Fig. 3a).

Building upon this, Lam Research also reported the thermal CVD of Sn-based resist thin films in 2019. The deposition of Sn-based resist materials was conducted at 70 °C and a process pressure of 2 Torr using metalorganic precursors, such as *t*-butyl tris(dimethylamino)tin and isopropyl tris(dimethylamino)tin, which react with an oxidant, such as water (H_2_O).^56^ During deposition, the water facilitates ligand–exchange reactions with alkylamino groups, while the unreacted alkyl ligands remain embedded within the film to participate in crosslinking upon EUV exposure (Fig. 3b). During EUV exposure and post-exposure bake (PEB), Sn–R bond cleavage forms a robust Sn–oxo network. This mechanism is similar to that of the PE-CVD Sn-based resist thin film system from the above, producing a dense inorganic network that enhances solubility contrast and etch resistance. Using 2-heptanone as the developer, the thermal CVD Sn-based resist thin films was able to resolve patterns with HP dimensions as small as 12 nm at an EUV exposure dose of 79 mJ cm^−2^.

### Molecular atomic layer deposition (MALD)

**Fig. 4:**
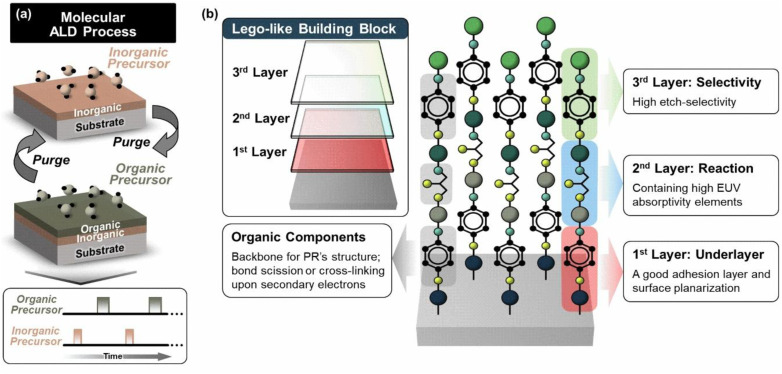
(a) Illustration of the MALD process for depositing hybrid organic–inorganic thin films using sequential pulse-purge cycles of organic and inorganic precursors. (b) Schematic of tunable, idealized vertically aligned multilayer architectures with precisely controlled chemical composition for high-performance hybrid EUV resists.

Although the synthesis of resist thin films *via* CVD shows great potential, ALD-based approaches offer unique opportunities and advantages for precise engineering of film structure and composition. As illustrated in Fig. 4a, a hybrid inorganic–organic resist can be constructed using a modified ALD approach, also known as molecular atomic layer deposition (MALD), in which inorganic and organic layers are periodically stacked with molecular-level precision.^39^ Incorporating metal-containing inorganic layers enhances EUV photon absorption, as metals exhibit much higher EUV absorption cross-sections than C and O in polymer-based resists, thereby minimizing photon loss.^30^ The organic layers, in turn, mediate secondary electron interactions that drive bond scission or cross-linking, either with metal centres to form metal–organic complexes or between adjacent molecular chains.^32^ Compared with solution-based approaches such as spin-coating, vacuum-based techniques provide atomic-scale control over thickness and composition, enabling the precise construction of multilayer architectures. For example, multilayer stack concepts can be derived from established EUV lithography patterning stacks, which consist of well-defined functional layers used in current process integration schemes. Fig. 4b illustrates an idealized vertically aligned multilayer architecture of the EUV resists; however, in practice, the actual films are expected to exhibit some degree of disorder due to random orientation of precursors during deposition.^57^ The proposed multilayer resist system may comprise an adhesion-promoting underlayer, a metal-containing imaging layer (*e.g.*, Sn-, In-, or Hf-based) with high EUV absorptivity, and a top layer designed to provide enhanced etch resistance for subsequent pattern transfer.^30,58^ Multia *et al.* highlighted the broad range of metal-containing and organic precursors available for the synthesis of hybrid films *via* MALD.^59^

In the following sections, we provide more detailed reviews of recent advances in hybrid inorganic–organic and metal–organic framework (MOF) resist thin films for EUV resist applications.

### Inorganic–organic hybrid resist thin films

Due to its high EUV cross-section absorption, Sn has attracted significant attention. In addition to the reported CVD Sn-based resist thin films developed by Lam Research, a group from Hanyang University (Republic of Korea) briefly demonstrated a MALD Sn-based inorganic–organic hybrid system deposited from tetrakis(dimethylamino)tin (TDMA-Sn) and ethylene glycol (EG) for EUV resist application.^60^ The Sn-EG resist thin film is formed through ligand exchange between the dimethylamino groups in the Sn precursor and hydroxyl groups (–OH) of EG.^61^ After exposure to DUV light, the Sn-EG hybrid resist system featured negative tone resist behaviour when developed in a diluted tetramethylammonium hydroxide (TMAH) solution. Material characterization using Fourier transform infrared (FTIR) spectroscopy and X-ray photoelectron spectroscopy (XPS) revealed that weak organic bonds like C–C, C–O, and C–H within the Sn-EG matrix were cleaved upon DUV irradiation. Simultaneously, oxidized metal bonds, such as Sn–OH and Sn–O, were formed, converting the material into a metal oxide-like network that is insoluble in low-concentration TMAH solution, thereby inducing negative-tone resist characteristic.

Beyond Sn, In has emerged as another promising candidate, owing to its similarly high EUV absorption.^62^ Lee *et al.* demonstrated an In-based MALD hybrid film (Indicone) using bis(trimethylsilyl)amidodiethyl indium (INCA-1) and hydroquinone (HQ) as a potential resist.^63^ electron beam irradiation induced a chemical transformation in the Indicone film, forming large graphitic carbon domains and causing indium loss, as confirmed by Raman and XPS. This is attributed to π–π stacking between benzene rings and the volatilization of In species. The chemical contrast between exposed and unexposed regions suggests that Indicone may function as either a positive or negative resist.

The Bent's group at Stanford University also reported the use of MALD inorganic–organic hybrid thin film composed of Hf and EG, successfully demonstrating negative-tone resist behaviour using 50 kV EBL patterning.^64^ This negative-tone hybrid resist exhibited a sensitivity (*D*_100_, defined as the minimum dose required to retain the full (100%) film thickness after development) of ∼400 µC cm^−2^ with a contrast value (*γ* = 1/log(*D*_100_/*D*_0_)) of ∼1.7 with *D*_0_ being the maximum dose at which the resist is fully removed, corresponding to 0% remaining film thickness. Using 3 M HCl as the developer, the Hf-EG resist resolved line patterns with a 50 nm half-pitch.

The group also investigated Al as the inorganic component in combination with EG, producing hybrid resist thin films that exhibited negative-tone characteristics similar to those reported by Ravi *et al.*^65,66^ The Al-EG system, however, displayed an approximately 45-fold decrease in sensitivity when evaluated using 50 kV EBL. Interestingly, oxygen-rich MALD Al-EG resist thin films have been reported to enhance sensitivity, reducing the required exposure dose from over 18 000 µC cm^−2^ to 4800 µC cm^−2^, although this remains roughly tenfold higher than the reported sensitivity for Hf-EG system. For both Hf-based and Al-based resist thin films, the solubility change upon electron beam exposure to metal oxide formation is expected to occur *via* C–O bond cleavage. Furthermore, it was suggested that the enhanced sensitivity in oxygen-rich Al-EG arises from pre-existing Al_2_O_3_ clusters, which require a lower dose to induce a solubility change in the diluted HCl developer solution. In contrast, the superior sensitivity of Hf-based resists was attributed to the greater oxophilicity of Hf, which makes HfO_2_ formation more thermodynamically favourable under exposure conditions. While these resist systems are promising for EUV resist application, the use of 3 M HCl as a developer limits their practical applicability for broader lithographic processes.

**Fig. 5:**
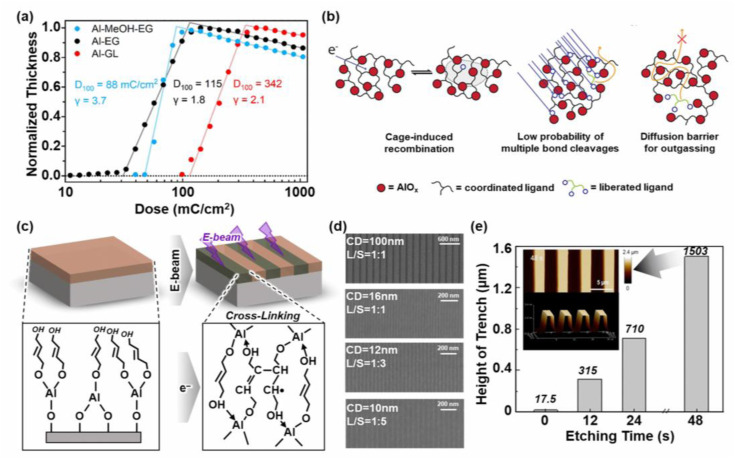
(a) Comparison of contrast curves for different organic ligands obtained using 100 kV EBL. (b) Schematic illustration of possible mechanisms where increased network density influences sensitivity.^67^ Copyright 2025, American Chemical Society. (c) Thin-film structure of TMA-BED resist and its proposed cross-linking mechanism under electron irradiation. (d) SEM images of high-resolution 50 keV EBL patterns of Al-based resists exposed at 40 000 µC cm^−2^. (e) Trench profiles on Si substrates after pattern transfer at varying etching times.^68^ Copyright 2024, Royal Society of Chemistry.

Furthermore, Than *et al.* recently reported the influence of network density on the patterning performance of various Al-based MALD hybrid resist thin films.^67^ In the study, resist films were synthesized using trimethyl aluminium (TMA) with different organic ligands, including ethylene glycol (EG), glycerol (GL), and subsequent methanol (MeOH)/EG dose. Among these systems, the Al-GL resist thin films showed the lowest sensitivity, with *D*_100_ of 342 000 µC cm^−2^, but exhibited an intermediate contrast (*γ* =2.1) compared to Al-EG (*D*_100_ = 115 000 µC cm^−2^, *γ* = 1.8) and Al–MeOH/EG (*D*_100_ = 88 000 µC cm^−2^, *γ* = 3.7), as shown in Fig. 5a. The authors attributed the reduced sensitivity observed for the Al-GL system to several factors that limit exposure-induced conversion efficiency. First, the increased coordination of glycerol ligands leads to a more densely cross-linked network, which can suppress secondary electron–driven reactions through a cage effect. In such an environment, reactive intermediates formed upon bond cleavage have limited spatial freedom and are more likely to undergo recombination rather than contribute to permanent chemical change. Second, because each glycerol ligand contains more hydroxyl groups than EG or MeOH, a greater number of bond cleavage events is required to fully detach a ligand and enable outgassing (Fig. 5b). Given that excitation events are more likely to be distributed across different ligands rather than repeatedly occurring at the same site, the probability of completely removing individual glycerol ligands is reduced. Lastly, the outgassing of organic fragments may be hindered by the higher network density, imposing a diffusion barrier that slows their escape. Together, these effects favour retention of the organic-rich network and impede the formation of inorganic nanoclusters during exposure, ultimately decreasing the resist sensitivity.

Instead of using a short-chain organic linker such as EG or GL, Wang *et al.* employed 2-butene-1,4-diol (BED), a longer organic molecule containing a C=C double bond, which is expected to enhance crosslinking reactions due to its high reactivity upon exposure.^68^ The resulting Al-BED resist exhibited a sensitivity of 450 µC cm^−2^ when subjected to 2 kV EBL using 10 wt% ammonia as the developer solution. FTIR and XPS analyses revealed that e-beam exposure resulted in a significant decrease in C=C bonds, a corresponding increase in C–C/C–H bonds, the formation of AlO_*x*_ species, and a slight reduction in C–O bonds, thereby elucidating the underlying exposure and crosslinking mechanisms. Density functional theory (DFT) calculations provided further insight into the lithographic mechanism of the Al-BED hybrid resist material. The calculation suggests that electron exposures induce C–H bond cleavage at the alkenyl site, *via* a transition state, followed by C–C coupling between neighbouring sp^2^ carbons, which has a low activation barrier of 0.19 eV. This process forms a cross-linked network responsible for the observed negative-tone resist characteristics (Fig. 5c). The Al-BED resist demonstrated excellent patterning capability, successfully resolving 10 nm line widths at 50 kV EBL (Fig. 5d). Remarkably, Fig. 5e shows its outstanding Si etch selectivity, reaching a minimum value of ∼86 with a trench depth of 1503 nm after 48 s etching, far exceeding that of conventional resists such as NMeBTA (2.3) and HSQ (6).^69,70^ These findings highlight Al-BED as a highly promising hard-mask resist for pattern transfer, although further improvements in sensitivity remain necessary.

**Fig. 6:**
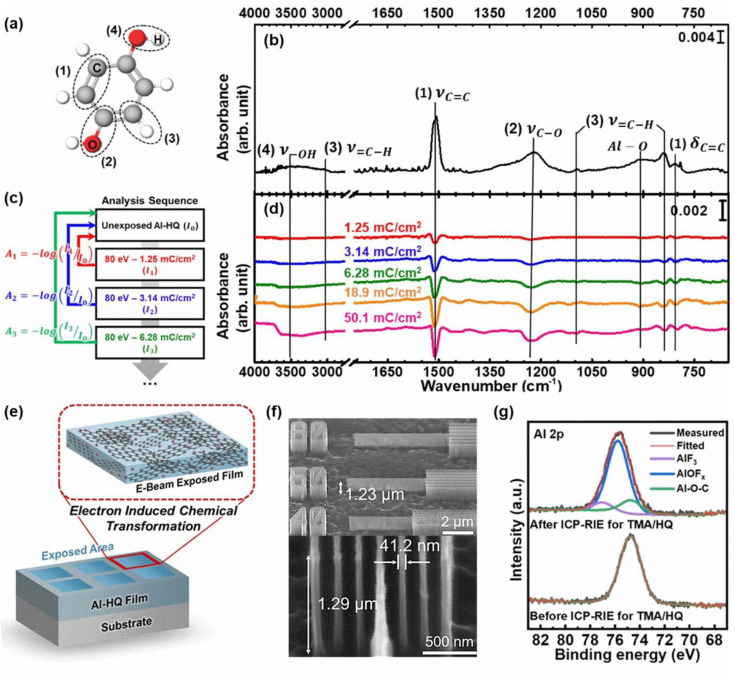
(a) IR-active bonds in the HQ molecule. (b) *Ex situ* FTIR absorbance spectrum of as-deposited, unexposed Al-HQ thin film, using HF-cleaned Si as reference. (c) Analysis workflow for obtaining absorbance spectra in the *in situ* FTIR study. (d) *In situ* FTIR spectra of Al-HQ under an 80 eV electron beam at different exposure doses. (e) Schematic illustration of chemical transformations induced by low-energy electron exposure.^71^ Copyright 2025, American Chemical Society. (f) Near-infinite Si etch selectivity using 30nm-thick Al-HQ resist. (g) XPS spectra for Al 2p core level before and after Si etch.^72^ Copyright 2026, John Wiley and Sons.

At the University of Texas at Dallas (UT Dallas), the Kim's group investigated MALD Al-HQ hybrid resist thin film for EUVL.^71^ The inorganic–organic hybrid resist thin films were deposited *via* ligand exchange reaction between TMA and HQ. When subjected to 0.1 kV EBL, the Al-HQ resist system exhibited a negative-tone characteristic with a sensitivity of 10 400 µC cm^−2^ and a reported contrast value of 5.71. The authors emphasized the use of *in situ* FTIR system equipped with an electron flood gun to monitor chemical changes in real time during low-energy electron exposure under ultra-high vacuum conditions, enabling precise identification of the reaction mechanism. The exposure energy of 92 eV was selected to simulate the interaction of EUV photons, while 80 eV was chosen as a representative energy for secondary electrons generated during EUV exposure, noting that the actual electron energy distribution depends on both valence- and core-level ionization processes. As illustrated in Fig. 6c, the absorbance was derived from single-beam spectra, revealing that low-energy electrons induced partial deformation or structural reorientation of HQ rings, evident from the dips at wavenumbers corresponding to aromatic (C=C, C–O, C–H, and O–H) vibrations (Fig. 6d). Complementary XPS and Raman analyses confirmed that exposure led to crosslinking between adjacent HQ molecules and the formation of graphitic carbon-like domains, which was also observed in the Al-4MP (4-mercaptophenol as linker) film system under the electron beam irradiation (Fig. 6e).^63^ The Al-HQ resist films also exhibited excellent etch resistance, enabling nearly infinite Si etch selectivity. Using only a 30 nm-thick Al-HQ as a hard mask, 40 nm-linewidth Si features with etch depths of 1.2–1.3 µm were achieved.^72^ This robust performance is attributed to the formation of an AlOF_*x*_ passivation layer under F-based etching conditions, which effectively preserves the integrity of nanoscale features (Fig. 6f and g). They also recently highlighted the critical role of the organic component, demonstrating a nearly 100-fold improvement in sensitivity for Hf-based negative-tone resist thin films when the organic linker was changed from 4 MP to DMP (2,3-dimercaptopropanol).^73^ Under 0.1 kV EBL, the sensitivities of Hf-DMP and Hf-4MP were found to be 76 and 2170 µC cm^−2^, respectively. This remarkable improvement in sensitivity is attributed to the distinct transformation pathways induced by low-energy electron irradiation. For resists containing aromatic linkers (*e.g.*, HQ or 4 MP), the exposure predominantly leads to the formation of graphitic carbon domains surrounded by metal oxides. In contrast, resists with aliphatic linkers undergo more complex transformations, where crosslinking occurs between metal centres and functional groups, resulting in a metal-core network that enables solubility switching.

**Fig. 7:**
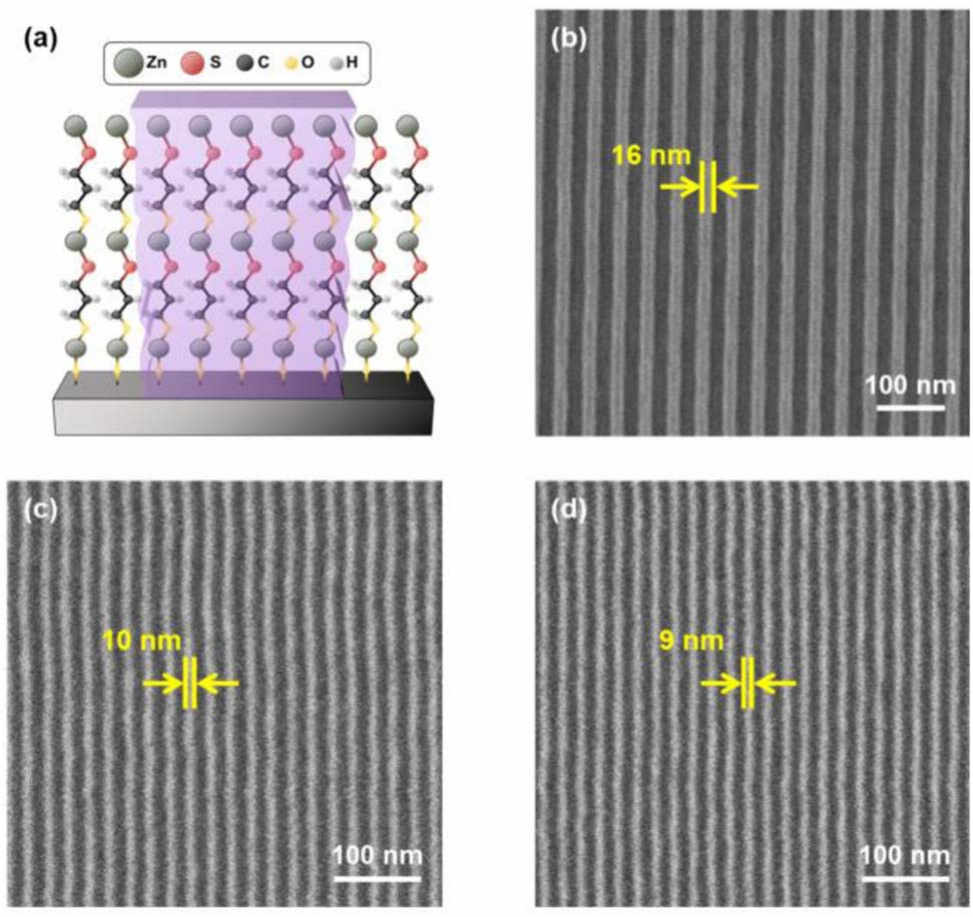
(a) Vertical molecular structure of the Zn-3MP hybrid resist. (b) 32 nm pitch line/space pattern resolved at a dose of 60 mJ cm^−2^. (c) 20 nm pitch line/space pattern, and (d) 18 nm pitch line/space pattern obtained using high-NA EUV tool. Reproduced with permission from ref. 75.

Zinc (Zn) is another inorganic component that is extensively studied in MALD hybrid resist thin films. With its two valence electrons available for bonding, Zn facilitates the formation of vertically oriented structures in MALD resist films, which is expected to help minimize LER due to the sub-nm lateral feature widths. The Sung's group at Hanyang University reported negative-tone Zn-based hybrid resist films formed from MALD process of diethylzinc (DEZ) and 3-mercaptopropanol (3 MP), where a vertically oriented wire-like structure was proposed (Fig. 7a).^74^ They found that *γ* of Zn-3MP was strongly influenced by development conditions. Optimizing developer concentration led to a significant enhancement in contrast, reaching *γ*_max_ = 6.46 with 0.35 wt% TMAH. The resist also exhibited uniform top-down development and consistently low surface roughness across varying remaining film thicknesses. Particularly, this system achieved an LER of only 1.37 nm for 16 nm half-pitch (HP) patterns at an EUV dose of 60 mJ cm^−2^, demonstrating high-resolution EUV lithography patterning capabilities (Fig. 7b). In addition, the study found that EUV exposure induced crosslinking reactions between neighbouring metal sites *via* the formation of Zn–O(S)–Zn coordination bonds between zinc atoms and O (S) atoms of functional groups. Recently, they demonstrated sub-10 nm HP patterning using a high-NA EUV exposure system at Lawrence Berkeley National Laboratory (LBNL, high-NA MET5). Notably, for 10 and 9 nm HP patterns, LER values of 1.49 and 1.34 nm were achieved, respectively (Fig. 7c and d).^75^

Other groups have also successfully realized Zn-based resist systems. For instance, Shan *et al.* reported a Zn-based resist system (Zn-EG) synthesized *via* an MALD process using DEZ and EG precursors.^76^ The resulting resist demonstrated successful pattern transfer on Si substrates, achieving 80 nm line resolution with a trench depth of approximately 14 nm. The exposure mechanism, investigated under 185 nm UV illumination, revealed notable chemical transformations. XPS analysis revealed oxidation reactions that converted C–H and C–O bonds to carbonyl (C=O) and carboxyl (O–C=O) groups, along with the formation of ZnO, as the mechanism of solubility switching. Meanwhile, Le *et al.* conducted a comprehensive study of the transformation mechanism in Zn-HQ hybrid resist thin films using *in situ* FTIR and Raman spectroscopy.^77^ They found that electron beam exposure induced crosslinking between benzene rings within the film, leading to the formation of graphitic carbon domains, which was consistent with observations in Al-based (Al-HQ and Al-4MP) and Hf-based (Hf-4MP) systems.^63,71,73^ Therefore, regardless of the metal species, hybrid resists with aromatic organic linkers tend to exhibit a similar lithographic mechanism. This mechanism likely explains the substantially higher dose required to induce a solubility change compared to aliphatic linkers. These results highlight the potential of hybrid inorganic–organic resists for next-generation high-NA and hyper-NA EUV lithography.

### Metal–organic framework thin films

Unlike hybrid inorganic–organic resist thin films, MOFs are porous coordination networks formed by the interactions between metal ions and organic linkers such as imidazolates or carboxylates *via* coordination bonds.^78^ Owing to their intrinsic porosity and tuneable chemistry, MOFs have been widely utilized in applications including gas storage, batteries, sensing, and catalysis.^78,79^ Recently, several studies have also shown the potential of MALD-based MOF thin films for EUV resist applications.

**Fig. 8:**
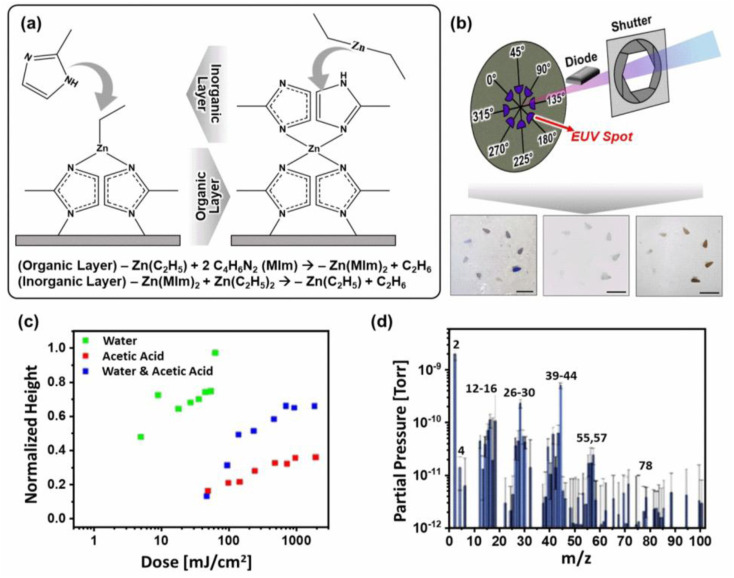
(a) MALD of amorphous zinc-imidazolate (aZnMIm) thin film. (b) Schematic of the EUV exposure setup, showing the shutter open and the diode rotated 135° out of the measurement position. (c) EUV contrast curves for aZnMIm resist developed by water, acetic acid, and combined water and acetic acid. (d) Outgassing spectrum during EUV exposure of aZnMIm resist.^81^ Copyright 2025, American Chemical Society.

The Tsapatsis' group at Johns Hopkins University reported a MALD Zn-imidazolate (aZnMIm) resist thin film fabricated by alternating DEZ and 2-methylimidazole (MIm) precursors.^80,81^ The film formed *via* proton–transfer reactions between DEZ and MIm, resulting in an amorphous ZnMIm coordination network where each Zn centre was tetrahedral coordinated to four imidazolate linkers (Fig. 8a).^80^ The sensitivity of the aZnMIm resist was evaluated using an EUV flood exposure, in which a diode was positioned between the sample and the EUV beam to monitor the beam current (Fig. 8b).^81^ During exposure, the wafer was rotated to a predetermined position, and the shutter was opened for a specific duration to deliver the desired dose. When developed in water, the films exhibited preferential removal of the 2-MIm component, but a residual Zn(OH)_*x*_-rich layer remained, despite the relatively high sensitivity of 5 mJ cm^−2^. In contrast, development in acetic acid rapidly dissolved both the exposed and unexposed regions, yielding no image contrast. Combining both water and acetic acid as the developer enabled residue-free patterning, though with a significantly reduced sensitivity of 181 mJ cm^−2^ (Fig. 8c). Outgassing analysis during EUV exposure (Fig. 8d) showed that aZnMIm primarily released H_2_, accompanied by weaker signals from small fragments of the 2-MIm ligand. Notably, aZnMIm can also function as an all-dry resist, enabled by a vapour-phase development process that will be discussed in the following section.

**Fig. 9:**
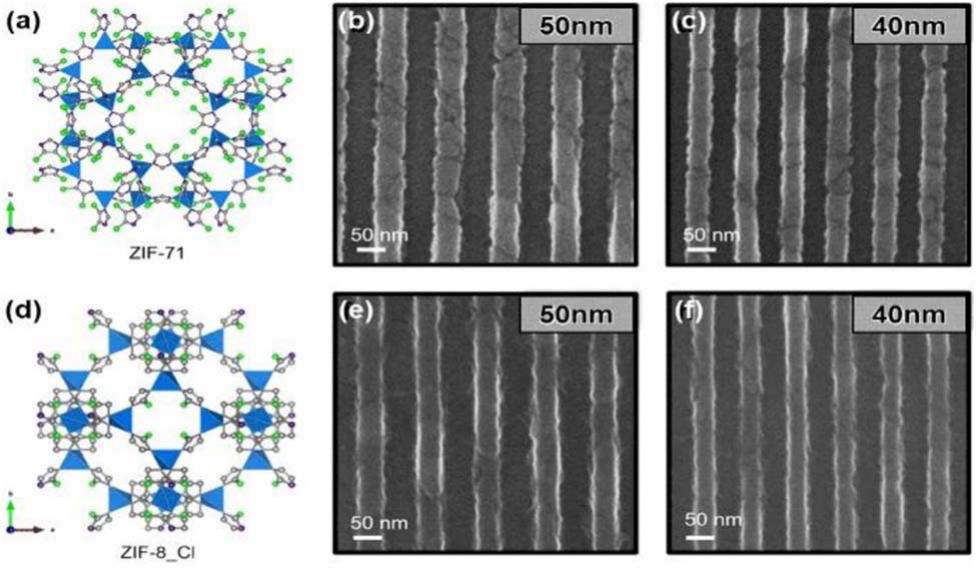
(a) Crystal structure of the ZIF-71 framework with 4,5-dichloroimidazole linkers. EUV lithography of 60 nm-thick ZIF-71 resist enables high-resolution patterning: (b) 50 nm half-pitch (HP) at 440 mJ cm^−2^ and (c) 40 nm HP at 425 mJ cm^−2^. (d) Crystal structure of the ZIF-8_Cl framework with 2-chloroimidazole linkers. EUV lithography of 80 nm-thick ZIF-8_Cl resist achieves (e) 50 nm HP at 400 mJ cm^−2^ and (f) 40 nm HP at 265 mJ cm^−2^, demonstrating enhanced sensitivity and fine feature definition.^84^ Copyright 2025, John Wiley and Sons.

Zeolitic Imidazolate frameworks (ZIFs), a class of MOFs, have recently shown potential as resist materials, with several reports demonstrating their e-beam patternability.^82–84^ Li *et al.* reported the patterning of halogenated ZIFs (ZIF-71 and ZIF-8_Cl) using EUV lithography, achieving a resolution of ∼40 nm at relatively high doses (Fig. 9).^84^ However, the resulting patterns exhibited poor LER, likely due to the crystalline nature and inherent surface roughness of the ZIF films.

In summary, MALD-based resists offer precise control over film composition and thickness, enabling uniform and tuneable architectures. While their sensitivity and stability still require improvement, advances in material design and process compatibility make them strong candidates for high- and hyper-NA EUV lithography.

### Secondary electron cascades in MALD-based hybrid resist thin films

**Fig. 10:**
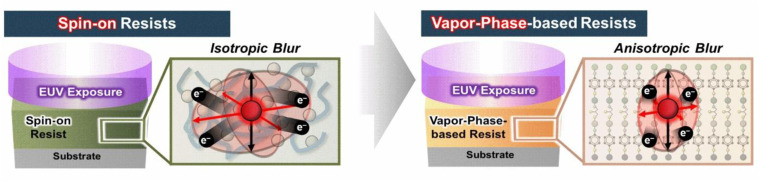
Comparison of absorbed-photon-induced secondary electron cascades: in spin-on resists, the heterogeneous matrix with discrete absorbing sites leads to longer mean free paths and isotropic blurring due to inconsistent reaction zones; whereas in vapor-phase-deposited films, the more uniform and densely packed structure is hypothesized to modify the effective electron transport behavior, which may contribute to more anisotropic (predominantly vertical, *z*-direction) charge delocalization and anisotropic blurring.

When the resist is exposed to EUV, the photoionization processes generate primary electrons with a distribution of kinetic energies (depending on valence- and core-level ionization pathways), and leave behind positively charged ions.^47^ These photoelectrons undergo elastic and inelastic scattering with nearby atoms or molecules.^45^ Inelastic scattering produces lower-energy secondary electrons, which drive most chemical transformations in the resist. The cascade and diffusion of these electrons, governed by their mean free path (*λ*), critically determine pattern resolution, as extended mean free paths lead to resist blur and increased LER.^48,85^ Resist blur is defined as the maximum spatial range from a photo-absorption event over which electrons propagate and drive chemical reactions that alter resist solubility, although in CARs and other PAG-based systems an additional contribution arises from photo-acid generation and subsequent acid diffusion, which further extends the effective spatial extent of the reaction.^86^ In conventional spin-on polymeric resists, EUV photons are absorbed within a heterogeneous matrix, and SEs scatter through irregular voids, creating broad lateral energy distributions and isotropic electron blur.^87^ Additionally, the resist blur in CARs is largely dominated by acid diffusion, which results in significantly higher LER, as the acid diffusion length (∼7 nm) is substantially longer than the electron scattering length (∼1–3 nm).^88,89^ In contrast, vapour-phase-deposited resists have higher density and reduced free volume, which shortens the SE mean free paths due to increased inelastic scattering, thereby suppressing lateral diffusion (Fig. 10).^90^ For example, according to the simulation conducted by Vaglio *et al.*, the average electron blur for CAR and Inpria MOR under EUV exposure is 1.5 nm and 1.1 nm, respectively.^91^ In addition, we hypothesize that when secondary electrons interact with a hybrid network comprising M–X–C linkages (where M denotes metal and X = O, S, or N) *via* electronic coupling, they can excite orbitals localized at the metal or X sites. These excitations may delocalize along the M–X bonds or the organic backbone, thereby enabling non-local bond dissociation or cross-linking, either vertically within the film or between neighbouring organic ligands (anisotropic blur along thickness (*z*)-direction).^92,93^ Long *et al.* demonstrated that increasing blur in the *z*-direction improves pattern uniformity and reduces dose-to-size without sacrificing resolution.^94^ Specifically, for common MORs, the critical linkage is M–O–C–R; where O 2p orbitals hybridize with metal d orbitals (in the M–O bond) and also weakly overlap with the hybrid orbitals of *R* (sp^2^ or sp^3^).^95^ SEs promote valence-to-conduction excitation-charge transfer from O 2p (+) to Metal d (−) orbital, potentially delocalizing over the network. Moreover, this charge transfer weakens the bonding character of M–O–C linkages, leading to bond dissociation (releasing organic fragments), crosslinking between adjacent M–O bonds (M–O–M crosslink), or oxidation/reduction.^96^ Therefore, for vapour-phase EUV resists with highly dense, ordered M–O–C bonding networks, charge-transfer excitation and delocalization can reduce localized overexposure and support uniform bond scission or cross-linking events across the film, thereby decreasing stochastic noise.

### Challenges associated with MALD-based hybrid resist thin films

Although MALD thin films have shown strong potential as a dry resist platform for EUVL, many of these systems exhibit instability under ambient conditions, leading to compositional or structural degradation over time, and limiting their practical applicability.

For instance, the thickness degradation of the Al-EG system upon exposure to ambient conditions has been reported by multiple studies.^97–99^ According to DFT calculations, resist films react with oxygen and water in the atmosphere, forming aldehydes or acids (reacted with oxygen) or glycols (reacted with water).^98^ Additionally, unreacted TMA ligands (–Al(CH_3_)) can be further oxidized, leading to unwanted oxide formation.^99^ Furthermore, the air stability of another Al-based systems has been investigated by various groups.^100,101^ Approximately 27% and 35% reduction in thickness observed for Al-HQ resist films upon air exposure and water dipping, respectively. It also found that the formation of carbonyl and carboxyl groups occurs from the reduction of –C–C/C–H bonds.

Similar behaviour was also observed in Zn-EG systems reported by Shan *et al.*, where the sample was exposed to air for an extended period, resulting in lower C–C/C–H concentrations that are expected to be converted to C=O.^76^ Meanwhile, Lewis *et al.* investigated the degradation mechanisms of zinc thiolate hybrid films, which are generally expected to exhibit improved stability owing to stronger Zn–S bonds compared to Zn–O bonds, which follows from hard and soft acid and base theory.^102^ Zn thiolate films prepared from two different dithiol linkers, 1,4-butanedithiol (BDT) and 1,2-ethanedithiol (EDT), were examined to elucidate their respective instability pathways. The authors observed that Zn-EDT films underwent substantial carbon loss, whereas Zn-BDT films showed more rapid sulphur reduction. They proposed that upon air exposure, reactions with moisture or oxygen cleave the C–S bonds in Zn-EDT, releasing small hydrocarbons, such as ethane, thereby reducing the carbon content. In contrast, sulphur loss in Zn-BDT was attributed to oxidation of the thiolate groups, followed by desorption as SO_*x*_ species. This differing behaviour may stem from packing density effects: the longer BDT linker promotes a more tightly packed coordination network, reducing the possibility of losing a large fragment like BDT. Although Zn thiolate films still exhibit week-scale degradation, their stability is notably improved relative to metalcone resists based on alcohol-derived linkers. Regarding lithographic performance after degradation, Zn-based thiolate exhibited a reduced contrast, consistent with the behaviour observed when water was intentionally introduced during film deposition.^103^ Thus, the lithographic performance of hybrid resists remains highly sensitive to moisture-driven chemical degradation, emphasizing the importance of more stable material designs for long-term reliability.

### VPI-derived resists and associated processes

VPI, a subclass of vapour-phase processing, enables the incorporation of inorganic elements, such as metals or oxides, into organic matrices, thereby enhancing EUV absorption owing to the higher absorption coefficients of metals compared with those of carbon and oxygen in polymer-based resists.^104^ Unlike MLD, which proceeds *via* self-limiting ligand–exchange reactions, VPI relies on sufficient precursor diffusion into the organic matrix to react with functional groups.^105^ Depending on the process sequence, VPI can be performed either before lithography, to tailor material sensitivity, etch resistance, and pattern fidelity, or after lithography, to improve etch selectivity during pattern transfer.

Baryshnikova *et al.* first introduced VPI to EUV lithography *via* sequential infiltration synthesis (SIS) of AlO_*x*_ into polymer-based EUV resist patterns (*i.e.*, VPI was applied after EUV pattering), reducing LER from 3.5 to 2.1 nm.^106^ However, this improvement was accompanied by film shrinkage and CD loss due to the removal of organic constituents.

**Fig. 11:**
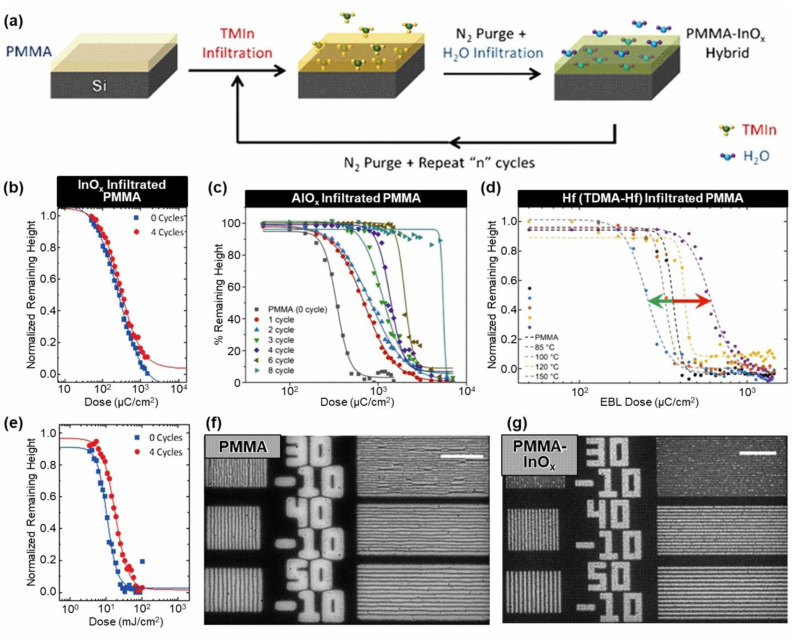
(a) Schematic of vapour-phase infiltration of InO_*x*_ into PMMA. Comparison of electron beam lithography (100 kV EBL) dose–response curves before and after infiltration: (b) InO_*x*_-infiltrated PMMA.^107^ Copyright 2023, John Wiley and Sons. (c) AlO_*x*_-infiltrated PMMA.^110^ Copyright 2019, Royal Society of Chemistry. (d) Dose curves of Hf (TDMA-Hf) infiltrated PMMA at various temperatures.^112^ Reproduced with permission from SPIE. (e) EUV exposure dose curves for pure PMMA and InO_*x*_-infiltrated PMMA. SEM images of line/space patterns by EUV lithography at 70 mJ cm^−2^ for (f) pure PMMA and (g) PMMA-InO_*x*_.^107^ Copyright 2023, John Wiley and Sons.

**Fig. 12:**
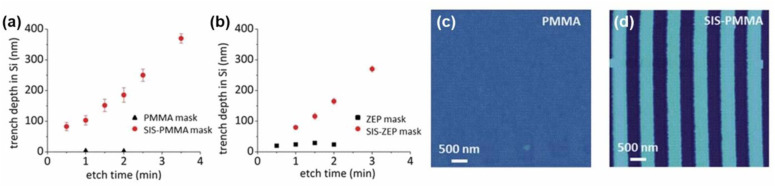
Effect of the VPI process on the etch resistance of PMMA and ZEP520A masks. Trench depths on Si substrates using (a) PMMA and infiltrated-PMMA masks, and (b) ZEP and infiltrated-ZEP masks. AFM topography images after 2 min of Si etching for (c) infiltrated PMMA and (d) PMMA mask.^119^ Copyright 2011, Royal Society of Chemistry.

It is the Nam's group at Brookhaven National Laboratory that first reported the inorganic-infiltrated organic-inorganic hybrid thin film for EUV lithography. They conducted a comprehensive investigation into the infiltration of various inorganic components into conventional PMMA resists. It was demonstrated that the formation of PMMA-InO_*x*_ hybrids *via* micro-dose VPI, where multiple precursor doses constituted one VPI half-cycle (Fig. 11a). The critical exposure doses for pristine PMMA and PMMA-InO_*x*_ were 250 and 325 µC cm^−2^ for 100 kV EBL and 10.2 and 17.6 mJ cm^−2^ for EUV lithography, respectively (Fig. 11b and e).^107,108^ In contrast, AlO_*x*_ infiltration resulted in a tenfold loss of sensitivity after eight cycles (Fig. 11c), attributed to the formation of a dense AlO_*x*_ layer near the surface that hindered PMMA removal at low exposure doses. This behavior likely originates from the high reactivity of TMA with carbonyl groups (–C=O) within PMMA, which forms a compact interfacial layer by rapidly reacting with PMMA surface groups, reducing porosity and limiting further AlO_*x*_ diffusion into the polymer matrix.^108–110^ Despite the observed sensitivity degradation, both InO_*x*_- and AlO_*x*_-infiltrated resists exhibited markedly enhanced etch resistance due to the incorporation of inorganic components. Using the PMMA-AlO_*x*_ hybrid as a hard mask, Si nanostructures with an aspect ratio of ∼17 (30 nm linewidth, 530 nm etch depth) were achieved, compared with an aspect ratio of ∼3–4 for InO_*x*_-infiltrated PMMA. Most recently, low-temperature Hf infiltration using only TDMA-Hf dosing was also shown to improve sensitivity, consistent with the findings previously reported by Hwang *et al.*, although the underlying mechanism remains under investigation (Fig. 12d).^111,112^ Compared to metal oxide infiltration in PMMA which can interfere with organic-solvent-based wet development process, TDMA-Hf-only infiltration introduces metallic species that enhance absorption without negatively affecting the wet development by organic solvent, thereby improving sensitivity. Regarding high-resolution patterning, pure PMMA showed poor pattern fidelity, with wavy lines and pattern collapse, whereas InO_*x*_ infiltration significantly improved line stability and pattern definition (Fig. 11f, g). Furthermore, improved sensitivity was demonstrated for a highly sensitive resist (HSR) *via* ZnO infiltration.^108^ Incorporation of ZnO into the positive-tone HSR matrix reduced the critical exposure dose compared to the unmodified resist infiltrated. For 100 kV EBL, *D*_50_ decreased from 338 to 171 µC cm^−2^. Similarly, under EUV exposure, the *D*_50_ reduced from 34 to 29.5 mJ cm^−2^. The HSR-ZnO system also showed excellent patterning capability, successfully resolving line features with CDs as small as 13 nm using 30 mJ cm^−2^. It is notable that the modification of material properties through infiltration has also been widely employed in various microelectronic applications, such as sensors and photodetectors, optoelectronic devices, and coatings.^113–117^

Meanwhile, post-lithographic infiltration of inorganic components has also been employed to improve etch selectivity, offering an effective approach to enhance etch resistance in ultra-thin resists designed for high- and hyper-NA EUV lithography. Unlike surface-only modification methods, VPI enables bulk hardening of the resist, protecting both the surface and pattern sidewalls during aggressive etching, thereby preserving LER. Tseng *et al.* demonstrated the SIS of AlO_*x*_ into patterns made of two common polymer-based resists, PMMA and ZEP520A (a copolymer of chloromethacrylate and methyl styrene), to enhance etch selectivity.^118,119^ The etch resistance increased by factors of 37 and 5 for infiltrated PMMA and ZEP520A, respectively, compared with the pristine resists (Fig. 12). This difference was attributed to the higher density of carbonyl (–C=O) groups in PMMA, which provide abundant reactive sites for TMA, whereas ZEP520A contains fewer such sites.^119^ LER changes were minimal, showing a slight decrease for PMMA and an increase for ZEP520A after SIS treatment. In addition, Vanelderen *et al.* reported a significant improvement in pattern LWR and LER after the transfer step, attributed to photoresist hardening that led to increased etch resistance.^120^

In summary, the effectiveness of the VPI approach relies on precise control of precursor chemistry, infiltration depth, and interfacial reactions, which together govern the balance between sensitivity, etch resistance, and pattern fidelity.

### Area-selective deposition (ASD) conjugated with lithography process

**Fig. 13:**
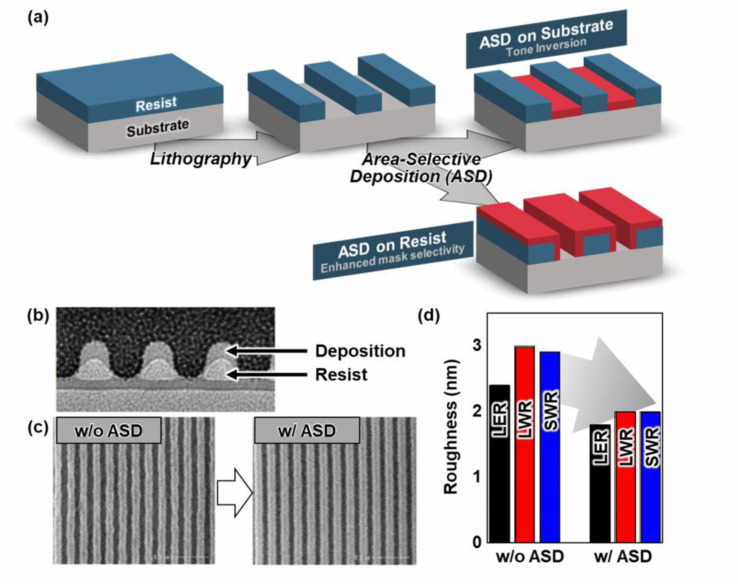
(a) Schematic of area-selective deposition (ASD) integrated with lithography, showing selective deposition on the substrate for tone inversion and on the resist to enhance etch resistance during pattern transfer. (b) TEM image highlighting ASD on the resist. (c) CD-SEM images demonstrating improved post-etch pattern fidelity with ASD compared to without ASD. (d) Roughness comparison, showing reduced roughness and enhanced pattern quality with ASD.^132^ Reproduced with permission from SPIE.

ASD is a vapour-phase technique that enables the controlled growth of thin films only on predefined regions of a substrate.^121^ It has become a key process in advanced semiconductor architectures, such as gate-all-around (GAA) transistors and three-dimensional dynamic random access memory (3D-DRAM), where selective oxide or metal gate deposition is required between different regions.^122–124^ In interconnect fabrication, ASD also facilitates selective metal filling and barrier layer formation.^125–127^ More recently, ASD has been explored in EUV lithography, either for tone inversion (ASD on substrate) or for selective deposition on resist films to enhance mask resistance (Fig. 13a).^128^

In particular, Nye *et al.* reported selective TiO_2_ deposition on polymer-based resists, with growth occurring on poly (*tert*-butyl methacrylate) (PtBuMA) but inhibited on poly(cyclohexyl methacrylate) (PCHMA). Notably, EUV-exposed poly(*p*-hydroxystyrene) (PHS) also suppressed TiO_2_ deposition. Thus, by carefully selecting the resist materials, ASD can enable either resist hardening or tone inversion.^128–130^

Meanwhile, other efforts have focused on ASD directly on exposed resist regions to locally harden the material, analogous to VPI or SIS, thereby improving pattern fidelity. Liu *et al.* investigated area-selective MALD (AS-MALD) of alucone from TMA and acetic acid (HAc) on PMMA.^131^ Unlike VPI, where AlO_*x*_ is distributed near the surface, AS-MALD uses a slow carrier gas flow during the diffusion step, enabling deeper precursor penetration. This approach yielded highly selective AlO_*x*_ deposition, with films at least 120 times thicker on PMMA than on Si-based substrates, attributed to –CH_3_ termination from HAc, which passivates the surface after a few cycles. The resulting alucone-PMMA exhibited high etch selectivities of 52 and 32 against SiON and SiO_2_, respectively. Similarly, Wada *et al.* further demonstrated improved pattern fidelity through the post-lithographic ASD process.^132^ Transmission electron microscopy (TEM) measurement revealed highly selective deposition on resist film rather than the substrate (Fig. 13b). The pattern with ASD exhibited reduced line waviness and an obvious reduction for all of the roughness metrics (LER, LWR, and side wall roughness (SWR)) (Fig. 13c and d). Meanwhile, Lutker-Lee *et al.* found that ASD not only preserves LER but also effectively heals line-break defects in thin-resist features, which is critical for high and hyper-NA EUV lithography.^133^ Another study, by Liu *et al.*, has also confirmed the promise of this approach, showing improved pattern roughness when integrated with the ASD process.^134^

Interestingly, in several reports, self-assembled monolayers (SAMs) have been explored as EUV-sensitive films that undergo chemical modification upon exposure, enabling subsequent ASD of etch masks. Lugier *et al.* demonstrated a bottom-up nanofabrication strategy using a fluorinated thiol SAM on Au, where EUV-induced chemical changes in the exposed regions promoted selective deposition of a surface-mounted MOF (SURMOF), which then served as a hard mask for Au etching.^135^ Remarkably, the required exposure dose was approximately 20–25 mJ cm^−2^, comparable to industrial EUV targets. Likewise, Lodha *et al.* reported EUV patterning of SAMs for selective Ru deposition; however, photoactive substrates (TiO_2_, SnO_2_, ZnO_2_) were required to catalyze SAM decomposition *via* photocatalysis, resulting in a higher dose requirement (∼500 mJ cm^−2^).^136^ While this approach is promising for meeting dose requirements (*e.g.*, SURFMOF), the achievable pattern resolution remains limited.

## Current progress on solvent-free vapour-phase development process for dry-deposited EUV resists

**Fig. 14:**
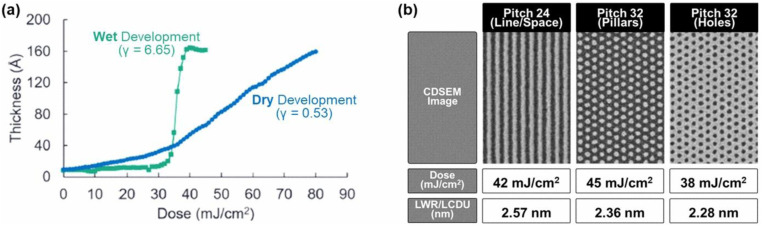
(a) Contrast curves under EUV exposure for CVD Sn-based oxide resist, comparing wet (light green) and dry (light blue) development. (b) High-resolution patterning of dense line/space features, pillars, and contact holes was achieved by dry development.^137^ Copyright 2024, The Society of Photopolymer Science and Technology (SPST), Japan.

Lam Research reported using HBr vapour as a chemical-vapour developer for Sn-based oxide resist films, enabling a fully dry patterning process with high throughput and eliminating pattern collapse issues inherent to capillary forces during wet development.^137,138^ In this approach, HBr selectively reacts with the unexposed regions to produce volatile RSnBr_3_ species. However, Fig. 14a shows a significant reduction in contrast from 6.65 (wet) to 0.53 (dry), due to the aggressive penetration of HBr, which reacts throughout the bulk and releases volatile RSnBr_3_ species. Notably, incorporating PEB together with optimized dry development conditions, specifically extended development time and elevated HBr pressure, significantly improves the contrast. As a result, Lam's dry resist achieved well-resolved 12 nm HP patterns with a 2.57 nm LWR at 42 mJ. cm^−2^ and an LCDU of 2.36 nm for 32 nm pitch pillars (Fig. 14b).

**Fig. 15:**
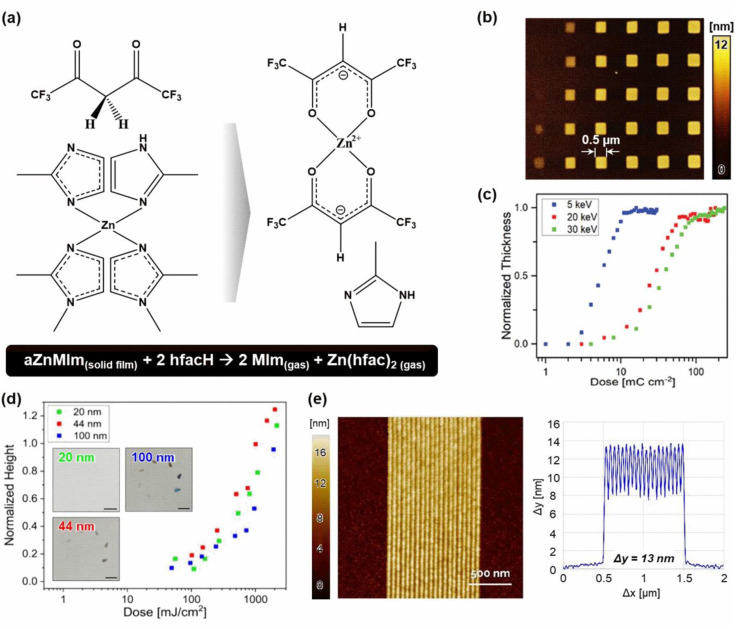
Dry development of MLD-based resists: (a) Dry etching of aZnMIm films using hfacH. (b) AFM image of the dose matrix for aZnMIm resist acquired at 5 keV EBL. (c) Exposure dose–response curves of aZnMIm at 5, 20, and 30 keV EBL, with normalized thicknesses determined from AFM analysis. (d) EUV contrast curves for dry-developed aZnMIm resists with different film thicknesses.^80^ Copyright 2024, John Wiley and Sons. (e) AFM image and corresponding thickness profile of vapour-phase-developed Zn4MP patterned with 10-nm lines at a 50-nm pitch.^139^ Reproduced with permission from SPIE.

**Fig. 16:**
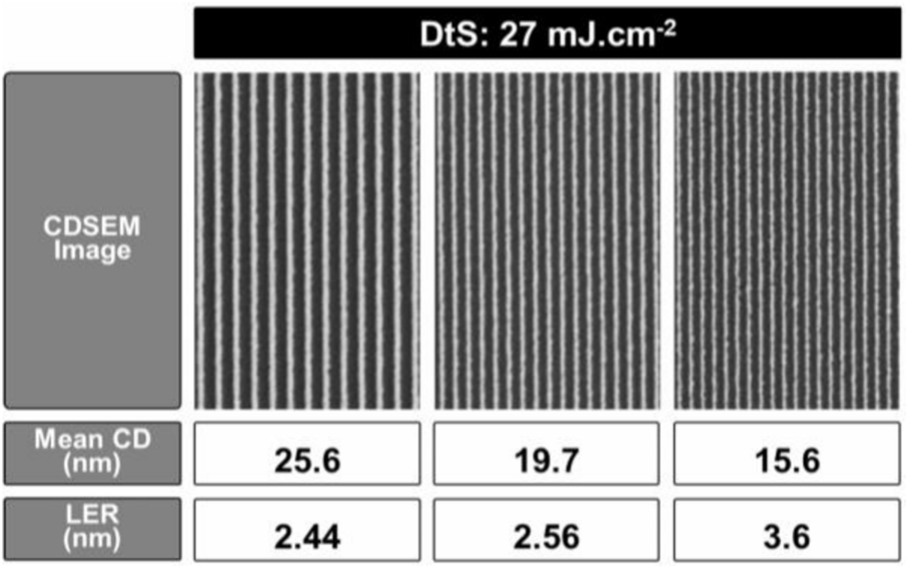
High-resolution line/space features obtained *via* dry development at a DtS of 27 mJ cm^−2^.^140^ Reproduced with permission from SPIE.

Corkery *et al.* and Seok *et al.* introduced hexafluoroacetylacetone (HfacH) as a chemical vapour developer for their Zn-based hybrid resist thin films.^80,139^ The ZnMIm resist film mentioned earlier could be developed using HfacH vapour, which selectively etched the unexposed regions by forming volatile Zn(hfac)_2_ complexes (Fig. 15a).^80^ The aZnMIm resist demonstrated sensitivities of 5.5, 28, and 37 000 µC cm^−2^ at 5, 20, and 30 kV electron beam lithography, respectively, and achieved well-resolved 22 nm features, which then decreased to 16 nm through the combination with appropriate underlayers. The reduced sensitivity at higher acceleration voltages was attributed to the lower probability of electron–matter interactions. Regarding the solubility switching mechanism, which is primarily associated with the degradation of the imidazolate linker, forming an amorphous carbon-nitrogen film containing Zn that is resistant to hfacH vapor. The resist was also evaluated by EUV exposure, reporting a sensitivity of approximately 375 mJ cm^−2^ with vapour-phase development, which remains relatively high for practical EUV lithography applications (Fig. 15d).^81^ The MALD-derived Zn-4MP hybrid resist also demonstrated compatibility with an all-dry patterning process. Upon electro-beam exposure, the films can be developed using HfacH vapour, yielding well-resolved features with a linewidth of approximately 10 nm (Fig. 15e).^139^ Similarly, Le *et al.* recently demonstrated an ultrasensitive hybrid resist that also enables an all-dry processing approach. At a low DtS of 27 mJ cm^−2^, features down to ∼15 nm were achieved with a LER of 3.6 nm (Fig. 16). Although further optimization is required to reduce LER, this resist system shows strong potential for improved dose efficiency, meeting the stringent dose requirements of EUV lithography.^140^

In addition to non-plasma thermal development, which relied on the formation of volatile reaction products between the gaseous developer and the resist film. Recently, the Yeom's group at Sungkyunkwan University proposed the use of Cl_2_ plasma to develop spin-coated Sn-based MORs.^141^ To minimize physical etching caused by high-energy ion bombardment in plasma, Cl radicals were delivered through a mesh grid positioned between the plasma source and the substrate. During dry development with Cl radicals, the unexposed regions were removed more rapidly due to the formation of volatile species, such as SnCl_*x*_, CO_*x*_, and C_*x*_H_*y*_, generated through Cl-induced bond scission. The unexposed regions exhibited a lower etching activation energy than the exposed regions; therefore, achieving high selectivity required operating at a reduced substrate temperature of −20 °C.

Although only a limited number of studies have demonstrated fully dry processing, these results underscore the potential of vapour-phase-processed resists as a scalable pathway for next-generation EUV lithography.

## Future and outlook

Looking forward, vapour-phase strategies present a promising pathway for next-generation EUV resists in high- and hyper-NA lithography by enabling precise molecular-level control over composition, thickness, and functional properties. These approaches open the door to designing ultrathin resist platforms with improved lithographic performance; however, further advances are needed to reduce exposure dose, suppress stochastic effects, and better understand exposure and development mechanisms. Recent progress in 3D-engineered dry resists, presenting vertically tailored EUV sensitivity profiles, offers a particularly promising direction.^142,143^ By introducing a sensitivity gradient across the film thickness, these systems help mitigate the intrinsic photon-absorption gradient in homogeneous resists, thereby improving depth uniformity of energy deposition. Such designs have been shown to enhance defect-free DOF window and reduce dose-to-size without compromising process latitude. In parallel, studies on MORs highlight the importance of process environment, where oxygen during post-exposure bake can significantly lower required dose by promoting ligand dissociation and Sn–O–Sn crosslinking.^144,145^ Overall, coordinated optimization of resist architecture and process conditions is expected to further improve performance for vapour-phase resists toward sub-10 nm patterning.

## Conclusions

As device dimensions continue to shrink with the transition from low-to high- and hyper-NA EUVL, achieving sub-10 nm patterning requires resist systems that simultaneously deliver high sensitivity, high resolution, and low LER. The continuous thinning of resists, necessary to match the reduced DOF, further challenges conventional solution-processed systems to maintain compositional uniformity and structural integrity. In this review, we have highlighted vapour-phase-based resist strategies as promising candidates for the next stage of EUV lithography, enabling exceptional film uniformity, higher density, and precise thickness control. This approach offers a versatile platform for designing multilayer resist architectures, where each layer serves a tailored function, such as adhesion promotion, EUV absorption, or etch resistance. A deeper understanding of EUV-induced secondary electron interactions in hybrid inorganic–organic systems will be crucial for optimizing sensitivity and pattern fidelity. Specifically, evidence from recent studies further supports our hypothesis that SE-induced charge transfers within M–O–C–R linkages can facilitate bond scission or crosslinking, influencing resist performance. Meanwhile, VPI has demonstrated improved pattern fidelity and etch selectivity by incorporating inorganic components into polymer matrices. Likewise, ASD has been effectively applied in conjunction with EUV lithography to enhance LER control and pattern transfer fidelity. Collectively, these developments establish a solid foundation for vapour-phase-engineered EUV resists. With continued mechanistic understanding and material innovation, these approaches are poised to advance the performance and scalability of next-generation lithographic materials for high- and hyper-NA EUV patterning.

## Author contributions

T. T. H. C. and D. N. L. and J. K. conceptualized and wrote the review. M. L. and T. T. H. C. prepared the figures. All authors contributed to the discussion of the content and approved the final version of the manuscript.

## Conflicts of interest

There are no conflicts to declare.

## Data Availability

No primary research results, software or code have been included and no new data were generated or analysed as part of this review.
